# Effect of Fermented Cottonseed Meal on the Lipid-Related Indices and Serum Metabolic Profiles in Broiler Chickens

**DOI:** 10.3390/ani9110930

**Published:** 2019-11-07

**Authors:** Jun-Li Niu, Jun Zhang, Lian-Qing Wei, Wen-Ju Zhang, Cun-Xi Nie

**Affiliations:** 1 College of Animal Science & Technology, Shihezi University, Shihezi 832003, China; niujunliaa@126.com (J.-L.N.); weilianqing_0524@163.com (L.-Q.W.); 2 State Key Laboratory of Animal Nutrition, College of Animal Science and Technology, China Agricultural University, Beijing 100193, China; June_zh16@cau.edu.cn

**Keywords:** fat deposition, metabolomics, cottonseed meal fermented by *Candida tropicalis*, white-feathered broiler

## Abstract

**Simple Summary:**

Excessive fat deposition in broiler chickens is detrimental for both producers and consumers. Fermented cottonseed meal (FCSM) was proposed as a potential method to relieve fat deposition in broilers’ rearing, while the physiological and metabolic mechanisms behind that still remain unclear. This study showed that both abdominal fat content and subcutaneous fat thickness significantly decreased in response to dietary FCSM supplementation at the age of 21 d, and altered the lipid-related metabolites index in serum, liver, and abdominal fat. Moreover, serum metabolic pathways were clustered into organic acid metabolism, fatty acid metabolism, and amino acid metabolism.

**Abstract:**

This study aimed to investigate the changes of lipid-related gene and serum metabolites in broiler chickens fed with fermented cottonseed meal (FCSM) diet, through quantitative real-time PCR and metabolomics analysis. Totally, 180 1-day-old Cobb broilers were randomly assigned to two groups with six replicates of 15 birds in each. The two diets consisted of a control diet supplemented with 0% FCSM (CON group) and an experimental diet with 6% FCSM (fermented by *Candida tropicalis*) replacing the soybean meal (FCSM group). The results showed that both abdominal fat content and subcutaneous fat thickness significantly reduced (*p* < 0.05) in response to dietary FCSM supplementation at the age of 21 d. Serum concentrations of glucose, triglyceride, and low-density lipoprotein cholesterol decreased (*p* < 0.05) in FCSM fed broilers compared with CON fed broilers, while the levels of epinephrine and growth hormone in serum, liver and abdominal fat tissue were higher (*p* < 0.05) in FCSM than in CON fed broilers. The activity of hormone-sensitive esterase and lipoprotein lipase (LPL) in the liver and abdominal fat were higher (*p* < 0.05) in FCSM than CON group. Additionally, compared with the CON group (*p* < 0.05), the expression of *peroxisome* *proliferator-activated receptor alpha* and *LPL* genes were upregulated in the livers of FCSM group broilers. Gene expressions of *hormone-sensitive lipase* and *LPL* in the abdominal fat tissue were also upregulated (*p* < 0.05) with the broilers fed with FCSM diets. A total of 20 significantly different metabolites were obtained in the serum of different dietary FCSM supplemented fed broilers. The mainly altered pathways were clustered into organic acid metabolism, fatty acid metabolism, and amino acid metabolism. These results not only provide a better understanding of broilers’ lipid metabolism with FCSM but also can be helpful in further improvement of the broilers’ healthy production and utilization of FCSM.

## 1. Introduction

It is well documented that the excessive intake of high-fat diets can lead to obesity [1], which is associated with health complications such as hypertension, dyslipidemia, and diabetes, or other adverse health conditions [2]. Therefore, over full-fat is an unpopular constituent of meat for consumers, being considered unhealthy.

The poultry population represents the largest number of livestock in the world, with relatively low feeding costs and high feed conversion ratio [3]. However, broiler breeds contain up to 13–14.5% [4,5] fat of their body weight, of which more than 85% of fat is not physiologically required. Excessive fat accumulation causes processing difficulties, but also indicates that feed energy use is inefficient [6] since the deposition one unit of fat consumes, is three times more energy than the deposition of one unit of lean meat [7]. Additionally, excessive fat deposition induces fatty liver, which also means both high mortality and incidence diseases in broilers [5,8]. Naturally, in summary, excess deposition of fat in broilers is detrimental for both producers and consumers.

For a long time, nutrition has an important effect on fat deposition, and nutrition regulation of broiler fat metabolism has been one of the topics concerned by researchers and broiler producers. Growing evidence demonstrates that the fermented cottonseed meal (FCSM) contributes to the lipid metabolism of broilers [7,9,10]. FCSM is produced by mixing solid cottonseed meal (CSM) with a certain amount of water, and then inoculating with beneficial microorganisms [11]. Microbial fermentation is currently considered as an especially promising way to reduce anti-nutritional factors in CSM [12,13], and FCSM is of improved nutritional composition compared with CSM [14]. The effects of FCSM on the lipid metabolism might be associated with the probiotics of FCSM [15,16,17] and the metabolites included in the fermented substrate, such as L-carnitine, small peptides, nicotinic acid, phosphorylcholine, amino acid, and organic acid [18]. However, further studies are needed to verify the effect of FCSM on fat deposition in broilers.

Metabolomics is a powerful method of describing the global metabolism of living organisms and capturing the metabolic changes associated with external stimulations [19]. Now, metabolomics has been used to study the lipid metabolism during fat deposition in pigs and chickens [10,20]. Therefore, this study aimed to assess the effects of FCSM on lipid-related genes, and serum metabolic changes in broilers using quantitative real-time PCR (qRT-PCR) and metabolomics analysis, and then attempted to explore the possible relationship among fat deposition, lipid-related genes expression, and serum metabolites.

## 2. Materials and Methods

### 2.1. Substrate Preparation and Fermentation

The strains of *Candida tropicalis* (*C. tropicalis*) were provided by the Feed Science Institute of Zhejiang University (Hangzhou, China). The fermentation was performed according to the process modified [13] protocol. For the fermentation of CSM, a substrate, containing 90% CSM, 5% wheat bran, and 5% corn flour, were mixed thoroughly with water in a ratio of 1:0.8 (wt:vol). The mixed substrate was then sterilized in an autoclave at 121 °C for 15 min. Then the substrate was taken out, cooled and each kilogram of substrate was mixed with 80 mL of *C. tropicalis* (1 × 10^8^ cells/mL). The mixture was fermented for 48 h in an incubator at 30 °C. After that, the substrate residue was dried in a drying cabinet at 40 °C for 48 h (dry matter content of FCSM is 91.6%). The dried substrate was ground and kept in the refrigerator pending diet preparation.

### 2.2. Animals and Experimental Design

All animal work in this paper was conducted according to relevant national guidelines. Animal care for the experiment complied with the regulations for the Animal Welfare Committee of Shihezi University (Xinjiang, China) (Ethical code: A2017-060-01). Totally, 180 1-day-old male Cobb broilers (500) were obtained from a commercial hatchery (Tengfei Poultry Industry Co., Ltd., Henan, China) and were randomly divided into two groups, which contained six replicates (15 birds each replicate). The birds in control group were fed with a soybean meal diet, and the experimental diet was supplemented with 6% FCSM replacing the equivalent amounts of soybean meal (according to our previous study [7]). The composition and nutrient levels of diets are shown in Table 1. The nutritional composition, fatty acid and amino acid profiles of FCSM and soybean meal are presented in Table 2. Broilers were raised in coops; each coop has three levels with five cages in each and three birds in each cage. Each cage was equipped with two nipple drinkers and one feeder. In order to fulfill their dietary needs, birds were offered and had free access to water during the whole trial period. The birds’ house was kept in 24 h constant light and room temperature was initially set at 33 °C, then gradually reduced to 25 °C by week 3. The experiment lasted 21 days (with protocol A2017-060-01).

### 2.3. Sampling, Data Collection, and Chemical Analyses

#### 2.3.1. Chemical Composition, Fatty Acid and Amino Acid Analyze of FCSM and Soybean Meal

FCSM by *C. tropicalis* and soybean meal from the experiment were analyzed for dry matter (DM), ether extract, crude protein and crude ash content following the standard method of AOAC (Association of Official Analytical Chemists) International [21]. Neutral detergent fiber and acid detergent fiber were determined using heat stable amylase and expressed inclusive of residual ash [22]. The contents of free gossypol were tested according to the standard method of the AOCS (American Oil Chemists Society) [23]. The samples for fatty acid profile analysis were prepared according to the National Standard of China (GB/T 21514-2008) and were analyzed with gas chromatography-mass spectrometry (ThermoFisher Trac1310 ISQ, Thermo Fisher Scientific Inc., Waltham, MA, USA). The amino acid profile was determined according to the National Standard of China (GB/T 18246-2000), as in a previous report [24].

#### 2.3.2. Growth Performance

The body weight and the feed intake of the experimental broilers were monitored weekly on a replicate basis. These data were used to calculate the average daily feed intake (ADFI), average daily gain (ADG) and feed-to-gain ratio (F/G; feed:gain). The F/G was calculated and corrected for mortality.

#### 2.3.3. Blood Samples

At the age of 21 days old, six broilers were randomly selected from each treatment (one bird from each replicate) and were sacrificed by cervical dislocation. All samples mentioned below were collected from the same six broilers (n = 6) except for histopathological sample (n = 4).

The blood samples were collected from the wing vein of birds after a 12 h feed withdrawal. In order to obtain serum, samples were then centrifuged at 3000× *g* for 15 min at 4 °C. All samples were dispensed and kept at −80 °C until further analysis. Serum samples were analyzed for the concentration of glucose (Glu), nonesterified free fatty acid (NEFA), total cholesterol (TC), triglyceride (TG), low-density lipoprotein cholesterol (LDL-C), high-density lipoprotein cholesterol (HDL-C), and the levels of epinephrine (EPI), growth hormone (GH), and insulin (INS).

The concentration of Glu, NEFA, TC, TG, LDL-C, and HDL-C were analyzed using Glucose Assay Kit (F006-1-1), Nonesterified Free Fatty Acids Assay Kit (A042-2-1), Total Cholesterol Assay Kit (A111-1-1), Triglyceride Assay Kit (A110-1-1), Low-density Lipoprotein Cholesterol Assay Kit (A113-1-1), and High-density Lipoprotein Cholesterol Assay Kit (A112-1-1), respectively (Nanjing Jiancheng Bioengineering Institute, Nanjing, China). The levels of EPI, GH, and INS were measured using Chicken Epinephrine ELISA Kit (DRE-C4601b), Chicken Growth Hormone ELISA Kit (DRE-C4308b), and Chicken Insulin ELISA Kit (DRE-C4306b), respectively (Kameishu Biotechnology Co., Ltd. Shanghai, China).

#### 2.3.4. Carcass Trait

At the age of 21 days old, six broilers were randomly selected from each treatment (one bird from each replicate) and were sacrificed by cervical dislocation. Abdominal fat was excised and weighed according to the description of report [25]. Meanwhile, according to the reported method [26], the thickness of subcutaneous fat (including the skin) in front of the caudal spondyles was determined using a Vernier caliper (precision is 0.02 mm). Data are presented as least squares means (LSM) ± standard error of means (SEM).

#### 2.3.5. Histopathological Analysis

Four broilers from each treatment were used for histopathological analysis. Abdominal fat was fixed in 4% paraformaldehyde. After being washed with water, all samples were dehydrated in gradient ethanol. Then they were made transparent in two changes of xylene for 10 min and 7 min, respectively. They were saturated and embedded with paraffin. The 5-μm-thick sections were stained with hematoxylin-eosin (H&E) and viewed under a light microscope (Leica DM LB2, Wetzlar, Germany) equipped with a fitted digital camera. All cells in three photos per slice for each bird were measured to obtain the adipocyte surface. Image analysis software Image-Pro Plus (Media Cybernetics, Inc., Bethesda, MD, USA) was used to measure the cross-sectional surface of the adipocytes. Data are presented as LSM ± SEM.

#### 2.3.6. Tissue Samples

After sacrifice, abdominal fat and liver tissues were collected from birds, immediately frozen in liquid nitrogen, and then stored at −80 °C for further analysis. Abdominal fat and liver samples were analyzed for the concentration of NEFA, TC, TG, and the activity or levels of hormone-sensitive lipase (HSL), malic enzyme (ME), acetyl CoA carboxylase (ACC), LPL, fatty acid synthase (FAS), EPI, GH, and INS. The concentration of NEFA, TC, TG, and the levels of EPI, GH, and INS were determined using the kits mentioned above. The activity of HSL, ME, ACC, LPL, FAS, EPI, GH, and INS were measured using Chicken Hormone-sensitive Lipase ELISA Kit (DRE-C9515b, Shanghai, China), Chicken Malic Enzyme ELISA Kit (DRE-C9600b, Shanghai, China), Chicken Acetyl CoA Carboxylase ELISA Kit (DRE-C9510b, Shanghai, China), Chicken Lipoprotein Lipase ELISA Kit (DRE-C9561b, Shanghai, China), and Chicken Fatty Acid Synthase ELISA Kit (DRE-C9527b, Shanghai, China), respectively (Kameishu Biotechnology Co., Ltd. Shanghai, China). Tissues samples and physiological saline were homogenized in a ratio of 1:9 (wt:vol), then the supernatant was taken by centrifugation at 3000× *g* for 15 min at 4 °C. All testing of samples was carried out according to the manufacturers’ protocol.

#### 2.3.7. qRT-PCR Analysis

Total RNA was isolated from the birds’ liver and abdominal fat with TRIzol reagent (Invitrogen, CA, USA). Reverse transcription of cDNA synthesis and qRT-PCR analysis were performed as described previously [7].

The gene primers of peroxisome proliferator-activated receptor-α, -γ (PPAR-α, -γ), ACC, FAS, LPL, and β-actin (internal reference gene) were obtained from previous study [7]. The gene primers of sterol-regulatory element-binding protein 1c (SREBP1c), HSL, and carnitine palmitoyltransferase-1 (CPT1) were designed in the GenBank (Table S2) and synthesized by BGI (Beijing, China).

#### 2.3.8. LC-MS/MS

The serum samples were analyzed by Water Acquity UPLC system (Suzhou Smartnuclide. Co. Ltd, Suzhou, China). The instrument equipped with an ACQUITY UPLC^®^ HSS T3 (150 × 2.1 mm, 1.8 μm, Waters, Milford, CT, USA) and the column was maintained at 40 °C. Autosampler temperature and flow rate were 4 °C and 0.25 mL/min, respectively. Gradient elution of analytes was performed with 0.1% formic acid water and 0.1% formic acid acetonitrile.

The ESI-MSn experiments were performed on the Thermo LTQ-Orbitrap XL mass spectrometer (Bremen, Germany). The spray voltage in positive ion is 4.8 and 4.5 kV in negative. The capillary temperature was 325 °C. The full scan was carried out with a resolution of 60,000 and mass range was 89–1000 m/z. Data dependent acquisition MS/MS experiments were performed with CID scan with a collision voltage of 30 eV. Dynamic exclusion was used to remove unnecessary MS/MS and the exclusion duration was set to 15 s.

The raw data were converted into the mzXML format using ProteoWizard software (http://proteowizard.sourceforge.net/; author, Chambers Mattew C; v3.0.8789) [27]. The R package XCMS (http://www.bioconductor.org/packages/release/bioc/html/xcms.html; author, Steffen Neumann; v1.48.0) was used for peaks area extraction, retention time correction, and peaks alignment [28]. The data of impurity peaks from column bleeds were deleted. The resulting two-dimensional matrix contained arbitrarily assigned peak indexes (retention time–m/z pairs), observations (samples), and ion intensity information (variables). The principal component analysis (PCA) and orthogonal projections to latent structures-discriminant analysis (OPLS-DA) were analyzed using the SIMCA 14.0 software (Umetrics, Umea, Sweden). PCA was used to visualize the clusters and display the similarity and difference. OPLS-DA was performed to further determine inter-group differences.

The significantly differential metabolites between two treatments were screened by the criteria with variable importance projection (VIP) > 1.0 and *p* < 0.05. The online databases, including METLIN (http://metlin.scripps.edu/) and KEGG (http://www.genome.jp/kegg/) were utilized to further identify and validate different metabolites. Identified differential metabolites between CON and FCSM-fed broilers were subjected to the MetaboAnalyst 3.0 software for metabolic pathway analysis (http://www.metaboanalyst.ca) [29].

#### 2.3.9. Correlations among Fat Deposition, Lipid-Related Indices, and Serum Metabolites

Serum metabolites, which VIP > 1.5 and *p* < 0.05, and main lipid-related indicators that were significantly altered by FCSM (*p* < 0.05) were used for interactive analysis using R (V3.2.4) software. Psych packages was used to calculated the correlations and *q* (false discovery rate, FDR) of Spearman’s rank. Correlations had an absolute Spearman’s correlation of > 0.50, with a *q* < 0.05. Pheatmap package (R) and omicshare tools website were used to visualize the correlations.

### 2.4. Statistical Analysis

Data were analyzed in the PROC MIXED procedure of SAS (SAS Institute Inc., Cary, NC, USA). R = 2^−ΔΔCt^ [30] was used to calculate the relative expression level of mRNA. The statistical unit of intake was coop, and the statistical unit of carcass, blood, and tissues was broiler. Least squares means and standard error of means were reported. Significant differences were declared at *p* < 0.05.

## 3. Results

### 3.1. Growth Performance and Fat Deposition

**Figure 1:**
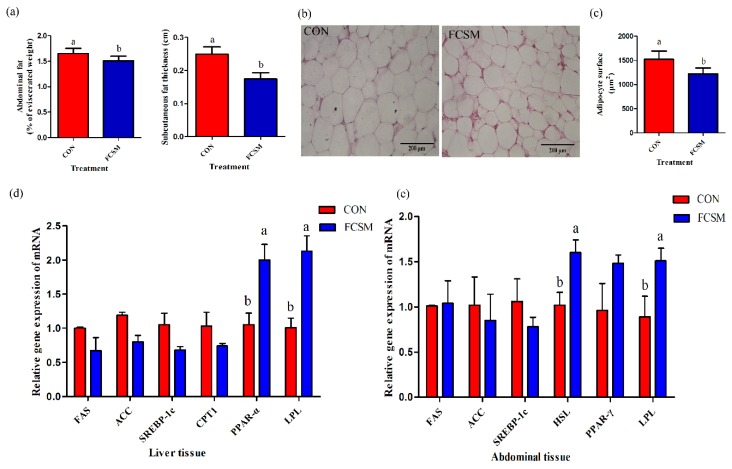
Fat deposition and lipid-related indices. ^a–b^ Means within a row with different superscripts differ significantly (*p* < 0.05). CON, no fermented cottonseed meal in diet; FCSM, 6% fermented cottonseed meal in diet. (**a**) Abdominal fat (% of eviscerated weight) and subcutaneous fat thickness (cm). (**b**) Adipocyte surface was demonstrated using hematoxylin-eosin (H&E) staining (Magnification = 100×; scale bar, 200 μm). (**c**) Mean abdominal fat size was estimated using the Image Pro-Plus software. (**d**) Lipid-related gene expression in the liver (*FAS, fatty acid synthase; ACC, acetyl CoA carboxylase; SREBP1c, sterol-regulatory element-binding protein 1c; CPT, carnitine palmitoyltransferase; PPAR-α, peroxisome proliferator-activated receptor-α; LPL, lipoprotein lipase*). (**e**) Lipid-related gene expression in the abdominal fat tissue (*FAS, fatty acid synthase; ACC, acetyl CoA carboxylase; SREBP1c, sterol-regulatory element-binding protein 1c; HSL, hormone-sensitive lipase; PPAR-γ, peroxisome proliferator-activated receptor-γ; LPL, lipoprotein lipase*). Error bars represent SEM.

Dietary FCSM induced the F/G (*p* = 0.04) without significant difference in body weight, ADFI, and ADG (*p* > 0.05) (Table 3). However, the percentage of abdominal fat (*p* = 0.03) and subcutaneous fat thickness (*p* = 0.04) were found decreased in FCSM treatment compared with the CON (Figure 1a). In addition, adipocyte surface was significantly lower (*p* < 0.01) for birds fed FCSM diet (Figure 1b,c). Histological analysis of adipocytes from abdominal fat showed significantly smaller adipocytes in FCSM (1221 ± 275 μm^2^, LSM ± SEM) broilers compared with CON (1524 ± 293 μm^2^, LSM ± SEM).

### 3.2. Lipid-Related Metabolites’ Indices in Serum, Liver, and Abdominal Fat

Ingestion of FCSM diet decreased the serum Glu (*p* = 0.04), TG (*p* < 0.01), and LDL-C concentration (*p* = 0.04). The concentrations of TC, NEFA, and HDL-C in serum did not change (*p* > 0.05) (Table 3). There were no significant alters of TG, TC, and NEFA in the liver and abdominal fat tissue, expect of TG in the liver (*p* < 0.01) (Table 4).

### 3.3. Lipid-Related Metabolites’ Enzyme Activity and Hormone Level in Serum, Liver, and Abdominal Fat

A higher EPI (*p* < 0.01) and GH (*p* < 0.01) concentration in serum were seen in FCSM group. Compared with CON group, broilers fed FCSM had lower levels of liver tissue ME (*p* = 0.01). Increased levels of liver and abdominal fat tissue HSL (*p* < 0.01, *p* = 0.01), LPL (*p* = 0.01, *p* < 0.01), EPI (*p* < 0.01, *p* < 0.01), and GH (*p* = 0.01, *p* < 0.01), respectively, could be observed in broilers fed with FCSM diet. There were no significant alters in the liver and abdominal fat tissue ACC, FAS, and serum INS levels between the two groups (*p* > 0.05) (Table 5).

### 3.4. Expression of mRNA in the Liver and Abdominal Fat Tissues

The gene expression of *PPAR-α* (*p* = 0.01) and *LPL* (*p* < 0.01) in the liver were clearly upregulated in birds fed the FCSM diet compared with CON diet. There were no significant alters of the mRNA expressions of *FAS*, *ACC*, *SREBP-1c*, and *CPT1* in the liver between the two groups (*p* > 0.05) (Figure 1d). In additional, the transcription levels of *LPL* (*p* = 0.03) and *HSL* (*p* = 0.01) were increased in the abdominal fat tissue of birds fed FCSM diet compared with CON group. The transcription levels of *FAS*, *ACC*, *SREBP-1c*, and *PPAR-γ* in the abdominal fat were not significantly affected by the addition of FCSM to the broiler diet (*p* > 0.05) (Figure 1e).

### 3.5. Metabolomics Profiling in Serum

The LC-MS/MS base peak chromatograms of serum from broilers fed two diets are shown in Figure S1. The PCA of LC-MS/MS data showed that two QC samples were tightly clustered (Figure S2), indicating that QC was repeatable and stable in the large-scale study.

**Figure 2:**
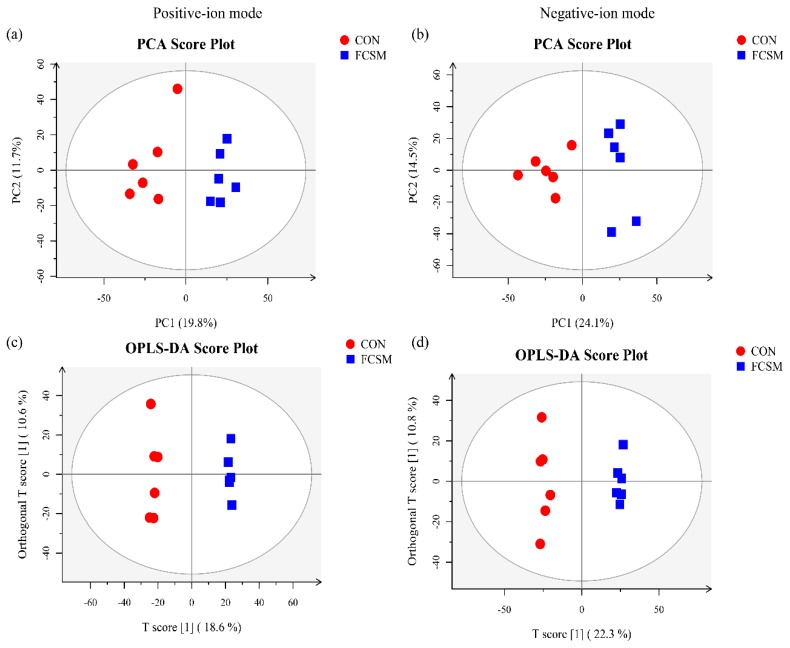
Principal component analysis (PCA), and orthogonal projections to latent structures-discriminant analysis (OPLS-DA) score plots analysis of LC-MS/MS data in positive-ion mode and negative-ion mode between CON and FCSM groups. CON, no fermented cottonseed meal in diet; FCSM, 6% fermented cottonseed meal in diet. (**a**) principal component analysis (PCA) in positive-ion mode. (**b**) Principal component analysis (PCA) in negative-ion mode. (**c**) OPLS-DA score plots in positive-ion mode. (**d**) OPLS-DA score plots in negative-ion mode.

The PCA analysis of LC-MS/MS metabolic profiles in broilers showed CON and FCSM groups were clearly separated clusters in both positive- and negative-ion modes (Figure 2a,b). The R^2^X values of the PCA in CON and FCSM groups were 0.525 (positive-ion modes), and 0.577 (negative-ion modes). The corresponding Q^2^Y values of PLS-DA models in CON vs. FCSM were 0.806 (positive-ion mode) and 0.852 (negative-ion mode) (Figure S3a,b). The OPLS-DA results are shown in Figure 2b,c. All the two treatments in serum score plots were within the 95% Hotelling T^2^ ellipse and separated clearly.

Totally, 20 significantly different metabolites (VIP > 1 and *p* < 0.05) were detected in the serum between the CON and FCSM diets (Table 6). Among them, seven metabolites belonged to organic acids including 2-Ethyl-2-Hydroxybutyric acid, 2-Hexenal, 3-Hydroxybutyric acid, azelaic acid, 4-Oxododecanedioic acid, salicylic acid, and trans-cinnamic acid; two metabolites were classified into amino acid; and each one metabolite was classified into peptides, lipids, pesticides, terpenoids, steroids, and alkaloids. In all the 20 significantly different metabolites, seven metabolites (3-Hydroxybutyric acid, 5-Methylthioadenosine, docosahexaenoic, biliverdin, dimethylglycine, prolylhydroxyproline, and 1-heptadecanoyl-sn-glycero-3-phosphocholine) had a higher concentration in the broilers fed with FCSM diet than the CON diet. Additionally, the concentrations of 13 significantly different metabolites (2-Coumarate, 2-Ethyl-2-Hydroxybutyric acid, 2-Hexenal, azelaic acid, 4-Oxododecanedioic acid, salicylic acid, N-acetyltryptophan, phenylethylamine, choline, costunolide, taurocholic acid, trans-Cinnamic acid, and indoleacetic) were significantly lower in FCSM-fed broilers than the broilers fed CON diet.

**Figure 3:**
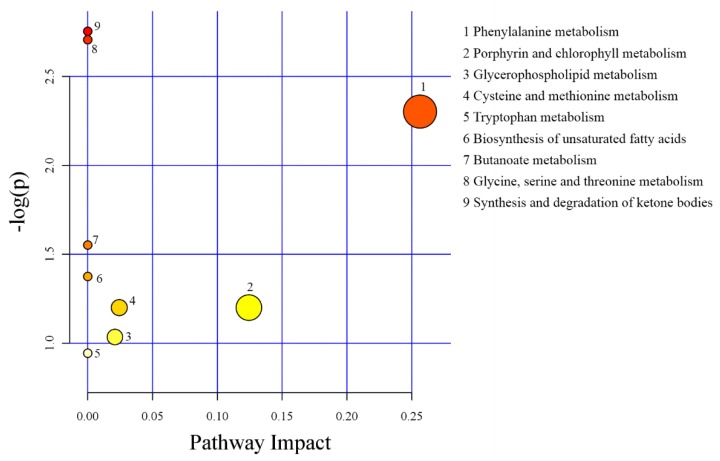
Pathway analysis of differential metabolites in serum between CON and FCSM groups using MetaboAnalyst 3.0. CON, no fermented cottonseed meal in diet; FCSM, 6% fermented cottonseed meal in diet. The colour of the circles from white to yellow to red denotes incremental fold change (−log(p)). The size of the circles from small to large indicates an increment of pathway impact.

Differential metabolites in serum among groups were analyzed by the MetaboAnalyst 3.0 and showed their related to metabolic pathways. Differential metabolites between CON and FCSM were involved in nine pathways, including synthesis and degradation of ketone bodies, glycine, serine and threonine metabolism, phenylalanine metabolism, butanoate metabolism, cysteine and methionine metabolism, porphyrin and chlorophyll metabolism, biosynthesis of unsaturated fatty acids, glycerophospholipid metabolism, and tryptophan metabolism (Figure 3).

### 3.6. Interactions of Serum Metabolites and Lipid-Related Indices

**Figure 4:**
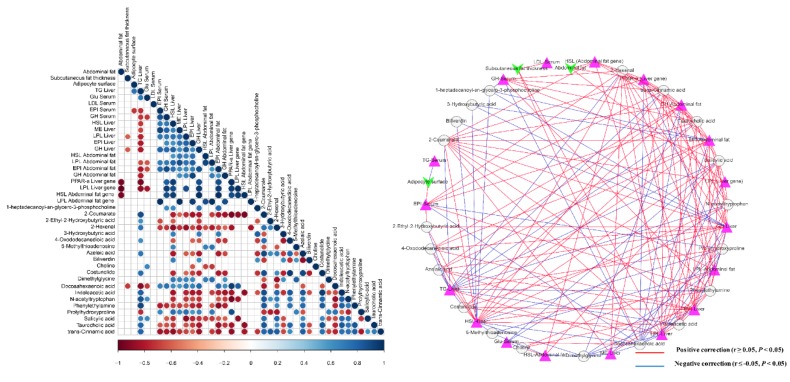
(The original image is presented in supplemental material). Correlation and co-occurrence network analysis among fat deposition, lipid-related indices, and predominant serum metabolites. (**a**) Correlation among fat deposition, lipid-related gene expression, and predominant serum metabolites. Correlation between fat deposition and lipid-related indices, and predominant serum metabolites. Cells are colored based on Spearman’s correlation coefficient: red represents a negative correlation, and blue represents a positive correlation. (**b**) Co-occurrence network analysis among fat deposition, lipid-related gene expression, and predominant serum metabolites. Each co-occurring pair among fat deposition, lipid-related gene, and serum metabolites has an absolute Spearman rank correlation above 0.55 [blue line, positive correlation (r ≥ 0.55); red line, negative correlation (r ≤ −0.55)] with a FDR-corrected significance level under 0.05. Fat deposition shown by green vee nodes, lipid-related indices are shown by pink triangle nodes, and metabolites are shown by gray round nodes.

Abdominal fat was negatively associated with *PPAR-α* (liver gene) (r = −0.94, *q* = 0.005), *LPL* (liver gene) (r = −0.99, *q* = 0.005), and *HSL* (abdominal fat gene) (r = −0.60, *q* = 0.005). Subcutaneous fat thickness was negatively related to LPL-liver (r = −0.64, *q* = 0.038), GH-liver (r = −0.67, *q* = 0.035), and docosahexaenoic acid (r = −0.74, *q* = 0.01). Adipocyte surface was positively associated with TG-serum (r = 0.74, *q* = 0.037), but it was negatively associated with EPI-serum (r = −0.71, *q* = 0.047). 3-Hydroxybutyric acid was positively associated with HSL-liver (r = 0.69, *q* = 0.047), GH-liver (r = 0.69, *q* = 0.013), and EPI-abdominal fat (r = 0.71, *q* = 0.01). Azelaic acid was positively associated with TG-liver (r = 0.73, *q* = 0.007), while negatively associated with HSL-liver (r = −0.81, *q* = 0.001), LPL-liver (r = −0.62, *q* = 0.03), EPI-liver (r = −0.62, *q* = 0.03), GH-liver (r = −0.83, *q* = 0.001), EPI-abdominal fat (r = −0.80, *q* = 0.002), and GH-abdominal fat (r = −0.66, *q* = 0.018). Biliverdin was positively associated with HSL-liver (r = 0.69, *q* = 0.014) and GH-liver (r = 0.60, *q* = 0.038). Docosahexaenoic acid was positively associated with EPI-serum (r = 0.68, *q* = 0.021), GH-serum (r = 0.75, *q* = 0.008), HSL-liver (r = 0.63, *q* = 0.028), ME-liver (r = 0.59, *q* = 0.045), LPL-liver (r = 0.72, *q* = 0.008), EPI-liver (r = 0.67, *q* = 0.017), GH-liver (r = 0.78, *q* = 0.003), EPI-abdominal fat (r = 0.76, *q* = 0.004), GH-abdominal fat (r = 0.62, *q* = 0.033), *PPAR-α* (liver gene) (r = 0.94, *q* = 0.005), and *LPL* (liver gene) (r = 0.83, *q* = 0.042), while negatively associated with TG-liver (r = −0.91, *q* = 0.001), and Glu-serum (r = −0.70, *q* = 0.016) (Figure 4).

## 4. Discussion

### 4.1. Growth Performance and Fat Deposition

From the present results, FCSM has no effect on birds’ growth performance (body weight, ADFI, and ADG), but markedly decreased the F/G, consistent with previous reports [17]. Free gossypol reduces the availability and digestibility of proteins in monogastric animals (especially poultry) by forming protein-gossypol compound in the CSM which inhibits the proteolytic activity and activities of digestive enzymes (pepsinogen, pepsin and trypsin) in poultry [31]. Fermentation of CSM with *C. tropicalis* greatly decreases free gossypol contents in CSM [13]. Improved growth performance (low F/G) may be associated with the elimination of the free gossypol.

Excessive fat accumulation in broilers is detrimental for both the broiler industry and consumers [7]. A large number of studies suggest that fermented food or feeds may be an effective strategy to regulating the fat deposition [10,32,33]. In the current study, we found that the content of abdominal fat and subcutaneous fat thickness were reduced in the FCSM group in comparison with the CON (Figure 1a). This stands in line with the findings of our previous research, which reported that the abdominal content fat was significantly reduced in the FCSM groups than in the control group [10]. The mechanisms underlying this FCSM reduced fat deposition are likely to be associated with the probiotics present in FCSM [15,16]. Furthermore, the reducing-fat deposition effect was also related to the metabolites (like amino acids, small-size peptides, vitamins) present in FCSM [18,34]. 85% of fat in a broiler’s body is not physiologically required. Abdominal fat accounts for 20% of total body fat, and is the chief site of fat deposition in broiler chickens [35]. The adipocyte surface of abdominal fat was smaller in FCSM-fed broilers in this study (Figure 1b,c). It appears, hence, that FCSM prevented adipocyte hypertrophy, thereby reducing fat accumulation.

### 4.2. Lipid-Related Metabolites’ Indices

Consistent with previous study results [36], broilers treated with FCSM supplementation had a lower in serum Glu and TG than the CON broilers (Table 4). Glu is one of the metabolites used as an indicator of the energy status of the animal [37]. In the present study, the concentration of Glu in broilers were measured after a 12 h feed withdrawal. The increased glucose concentration in CON indicates that it is probable that FCSM alleviates fat deposition by affecting energy metabolism in chickens. TG are made of a glycerol and fatty acids, which comprise almost all ingested fat [38]. The TG is stored in lipid droplets, which is the main component of serum lipids [39]. Serum lipids are circulated in the blood by being encapsulated in lipoproteins [40]. HSL form a class of lipoproteins, and function as a cleaner that transports fatty acids and cholesterol from the tissue to the liver, hence considered a “good” cholesterol [16]. Otherwise, LDL play a negative role in serum cholesterol transportation, because LDL are vulnerable to reactive oxygen species, forming an oxidized ox-LDL, which is considered to be a major risk factor for atherosclerotic disease [41]. Interestingly, the decrease in the level of LDL-C in FCSM-fed broilers found in our study was significant, even though the FCSM did not change the level of HDL-C and TC. Serum LDL is the most important cholesterol-carrying lipoprotein [42]. It is, therefore, suggested that intake of FCSM did not decrease serum TC but modified serum cholesterol composition through a reduced LDL-C level.

Our results showed increased levels of GH in the FCSM group compared to the CON group. GH can activate lipids as well as inhibit the synthesis of TG. For example, GH downregulates the gene expression of *LPL*, upregulates *HSL* and uncoupling proteins, improving the sensitivity of fat tissue to adrenergic stimulus as well as reducing the transportation of Glu [43]. In a study of elderly obese rats, results showed that feeding GH to animals assisted in breakdown of fat tissue and a short-lived decrease in voluntary food ingested leading to body weight loss [44]. Another study suggested that supplementation of GH assisted in weight loss, increased fat breakdown as well as oxidation in genetically-obese mice [43]. These results from our study indicated that FCSM might decrease fat deposition in broilers through improving the levels of serum EPI and GH, as well as the levels in the liver and abdominal fat.

The activity of LPL, a lipogenic enzyme that regulates adipocyte lipid uptake [45], was increased in the abdominal fat and liver tissue in FCSM group broilers. Differently from mammals, the lipids deposited in the fat tissues of birds are mainly synthesized in the liver and absorbed by the LPL [25]. LPL was considered to be a lipase responsible for TG hydrolysis, since there is a direct relationship between LPL activity, and TG breakdown existed under different conditions [46]. Thus, it is probable that fat deposition reduction effect is related to the increase of LPL, in the current study.

HSL, an adipocyte enzyme that lyses fatty acids from intracellular TG [47], is regarded as a rate-limiting enzyme for lipolysis in animal fat tissue [48]. Our results suggest that the activity of HSL increased in FCSM group, which was opposite to that of abdominal fat content. Correlation results suggest that the alter of abdominal fat content might be partially due to the alter of HSL activity in the abdominal fat.

In the present trial, the supplementation of FCSM enhanced the levels of EPI in serum and liver, as well as abdominal fat tissues. Elevated EPI levels caused TG breakdown through stimulation of HSL [49]. It seems that the EPI-induced HSL activation is mediated by *β*-adrenergic activation of protein kinase A, which phosphorylates and activates the enzyme [39]. Although further research is needed on this issue, the current result shows that the decreased TG concentration and the increased HSL is associated with elevated EPI levels in broiler chickens.

### 4.3. Lipid-Related mRNA Expression

Meanwhile, FCSM addition upregulated the gene expression of lipid-related metabolism in broilers. *PPAR-α* is highly expressed in the liver and plays a key role in regulating Glu and lipid metabolism or enhancing the gene expression involved in fatty acid oxidation [50,51,52]. The mRNA expression of *PPARα*, increased in broilers fed with FCSM, was consistent with our team’s previous finding, that intake of FCSM improved *PPARα* mRNA expression in the liver compared to CON group [7]. These results showed that added FCSM to the diet could decrease the fat deposition by enhancing long-chain fatty acids oxidation in mitochondria and fatty acid oxidation in the liver [53,54]. LPL, is synthesized mainly in fat tissues and skeletal muscles, can hydrolyze TG of chylomicrons and LDLs to free fatty acid and glycerol, which go into fat tissue and are esterified into TG [39]. In adipose tissues and liver tissues, higher gene expression of *LPL* in broilers fed with FCSM diet indicates that FCSM may improve lipolysis [55,56]. Compared with the CON, broilers fed with FCSM had significant increase in the transcription of fat *HSL*. These data suggested that FCSM might relieve the fat deposition in the liver and abdominal fat tissue by upregulating the mRNA expression associated with fatty acid oxidation.

### 4.4. Metabolomics Profiling in Serum

Metabolomics is a forceful method to describe the global metabolism of living organisms and capturing the metabolic changes associated with external stimulations [19]. Now, metabolomics has been used to investigate the lipid metabolism during fat deposition in animals [20]. This study reveals the changes of the lipid metabolism in FCSM-fed broilers by comprehensive analysis of metabolites altered in serum. Several metabolites involved in the serum metabolism were different between CON and FCSM group. The present results provided a new perspective for the distinguished metabolomics profile and might be used to investigate the differences in lipid metabolism between broilers fed with CON and FCSM diet. In our study, among the significantly changed metabolites, most of them were organic acids, such as 2-ethyl-2-hydroxybutyric acid, 2-hexenal, 3-hydroxybutyric acid, azelaic acid, 4-oxododecanedioic acid, and salicylic acid. Additionally, some were amino acid, peptides, and lipid.

Ketone bodies, mainly produced in the liver of animals by fatty acid oxidation, can be used as alternative energy sources in some cases [57,58]. The metabolism of ketone bodies is related to sterol biosynthesis, *β*-oxidation and de novo synthesis of fatty acids as well as intracellular signal transduction [59]. 3-hydroxybutyric acid is a small lipid-derived molecule metabolite, which is collectively referred to as a ketone body with acetone and acetoacetic acid [60]. 3-hydroxybutyric acid can be served as an energy source when Glu is low [50]. In the current study, the concentration of 3-hydroxybutyric acid increased in FCSM group than in CON group. Moreover, 3-hydroxybutyric acid was positively associated with HSL and GH in the liver, and EPI in the abdominal fat (Figure 4), which illustrated that 3-hydroxybutyric acid may have affected fat deposition through the HSL, GH, and EPI in broilers fed with FCSM.

Azelaic acid, a saturated 9-carbon atom dicarboxylic acid, in urine is usually used to indicate the fatty acid oxidation [61,62]. It has been generally thought that azelaic acid is produced by *ω*-oxidation of long-chain unsaturated fatty acids followed by their *β*-oxidation [63]. FCSM resulted in increased effects on azelaic acid, indicating that FCSM enhanced the fatty acid oxidative metabolism in broilers. Interestingly, azelaic acid was negatively associate with serum TG, and positively with liver HSL, LPL, EPI, GH, and abdominal EPI and GH.

Additionally, higher relative content of biliverdin was observed in the FCSM group compared to CON group in the present study. Recently, it was reported that biliverdin has a strong anti-lipogenic effect [64]. More specifically, at the higher doses, biliverdin reduced lipid accumulation by 91%, and upregulated *PPAR-α* target genes. Thus, these results suggested that FCSM may reduce the fat deposition in broiler by increase the serum biliverdin levels and thereby upregulated the expression of *PPAR-α* genes. Furthermore, biliverdin is a scavenger of reactive oxygen species and can provide cytoprotection against cellular oxidative injuries [65]. Additionally, it is well known that reactive oxygen species can generate during the process of fat oxidation. Therefore, we suspected that the increase of biliverdin in this test is related to the acceleration of fat oxidation.

Polyunsaturated fatty acids, specifically docosahexaenoic acid, are associated with promoted fatty acid oxidation [66], and decreased fatty acid synthase gene expression as well as inhibition of de novo fatty acid synthesis [67]. Meanwhile, it was reported that the expenditure of 60% of dietary energy as *ω*-3 fatty acids improved uncoupling protein 2 in liver and prevented obesity in mice [68]. Conversely, the diet rich in polyunsaturated fatty acids results in the alteration of the metabolism of adipose tissue that is beneficial for fat deposition [66]. In our study, FCSM presented higher polyunsaturated fatty acid content than soybean meal. Additionally, LC-MS/MS data showed that docosahexaenoic acid concentration increased in FCSM-fed broiler serum than in CON. And docosahexaenoic acid was negatively associated with subcutaneous fat thickness, serum TG, and Glu, and positively associated to the enzyme activity and hormone levels that promotes lipid-mobilization in broiler liver and abdominal fat tissue. Thus, although this issue needs further investigation, our study revealed that the low-abdominal fat effects of FCSM might be related to the higher docosahexaenoic acid in the blood.

## 5. Conclusions

In our study, serum metabolomics method was used to investigate the main altered lipid metabolic process changing associated fat deposition FCSM-supplement diet fed broilers. Broilers supplemented with FCSM presented lower abdominal fat, subcutaneous fat thickness and small adipocytes surface at the age of 21 d. The lipid-related enzyme and hormone in the serum, abdominal fat, and liver tissues, as well as the gene expression in abdominal fat and liver tissues, indicated that the broilers fed with FCSM-diet had higher fatty acid oxidation. Based on the metabolomics analysis, we found that the organic acid metabolism, fatty acid metabolism, and amino acids metabolism were the main different pathways. The understanding of these processes can lead to a better application of the FCSM in the broilers’ breeding.

## Figures and Tables

**Table 1:** Diet compositions and nutrient contents of the experimental diets ^1^.

Item	CON	FCSM
Ingredients, %DM		
Yellow corn	54.50	54.30
Soybean meal	33.50	27.40
Fermented cottonseed meal	0.00	6.00
Sunflower oil	3.00	3.30
Cottonseed protein	4.00	4.00
Premix ^2^	5.00	5.00
Chemical compositions		
Metabolizable energy, Mcal/kg	2.95	2.95
Crude protein, % of DM	21.23	21.23
Ether extract, % of DM	5.8	5.5
Crude fiber, % of DM	2.4	2.5
Calcium, % of DM	1.04	1.03
Total phosphorus, % of DM	0.69	0.71
Available phosphate, % of DM	0.45	0.45
Methionine, % of DM	0.50	0.50
Methionine + cysteine, % of DM	0.86	0.86
Threonine, % of DM	0.78	0.76
Lysine, % of DM	1.12	1.08

^1^ CON, no fermented cottonseed meal in diet; FCSM, 6% fermented cottonseed meal in diet. ^2^ Premix provided the following per kg of diets: Ca 8.106 g/kg; P 2.98 g/kg; Nacl 2.94 g/kg; Lys 0.234 g/kg; Met 1.791 g/kg; VA 8800 IU/kg; VD 3000 IU/kg; VE 30 mg/kg; VK_2_ 1.65 mg/kg; VB_1_ 2.5 mg/kg; VB_2_ 6.6 mg/kg; VB_3_ 11 mg/kg; VB_4_ 500 mg/kg; VB_5_ 60 mg/kg; VB_6_ 4.0 mg/kg; VH 0.2 mg/kg; VB_11_ 1.0 mg/kg; VB_12_ 0.02 mg/kg; VC 50 mg/kg; Fe^2+^ 80.0 mg/kg; Cu^2+^ 8.0 mg/kg; Zn^2+^ 60.0 mg/kg; Mn^2+^ 70.0 mg/kg; I 0.5 mg/kg; Se^4+^ 0.3 mg/kg.

**Table 2:** Chemical composition, fatty acid and amino acid profile of FCSM and soybean meal ^1^.

Item	FCSM	Soybean Meal
Chemical Composition		
Dry matter, %	91.26	90.52
Crude protein, % of DM	44.42	44.26
Ether extract, % of DM	0.86	1.87
Crude ash, % of DM	6.43	6.24
Acid detergent fiber, % of DM	13.08	10.21
Neutral detergent fiber, % of DM	21.78	13.52
Free gossypol, mg/kg	36.41	-
Fatty Acid, %		
C14:0	0.0069	0.0015
C16:0	0.2304	0.1255
C18:0	0.0341	0.0405
C20:0	0.0048	-
C22:0	0.0056	0.0074
C24:0	0.0037	0.0027
Saturated fatty acids	0.2855	0.1775
C16:1	0.0047	0.0007
*cis*-C18:1n-9	0.2265	0.1211
C20:1	0.0015	-
C22:1n-9	0.0308	0.0268
Monounsaturated fatty acids	0.2636	0.1486
*cis*-C18:2n-6	0.3280	0.2566
C18:3n-3	0.0061	0.0339
C22:2	0.0010	0.0009
Polyunsaturated fatty acids	0.3351	0.2914
Amino Acid, %		
Aspartic acid	4.12	5.60
Threonine	1.55	2.05
Serine	2.07	2.59
Glutamate	9.63	9.26
Glycine	1.86	2.09
Alanine	1.84	2.25
Cystine	0.68	0.67
Valine	2.03	2.30
Methionine	0.35	0.54
Isoleucine	1.27	2.09
Leucine	2.63	3.87
Tyrosine	0.89	1.48
Phenylalanine	2.54	2.62
Lysine	2.05	3.12
Histidine	1.21	1.22
Arginine	4.99	3.55
Proline	1.91	2.66

^1^ FCSM, fermented cottonseed meal in diet.

**Table 3:** Effect of FCSM on the growth performance of broiler.

Items ^1^	Treatment ^2^		SEM ^3^	*p*-Value
	CON	FCSM		
Body weight, g	596.67	581.53	14.53	0.63
ADFI, g/d	65.61	59.51	0.73	0.33
ADG, g/d	43.80	42.09	0.87	0.49
F/G, g/g	1.53 ^a^	1.41 ^b^	0.06	0.04

^1^ ADFI, average daily feed intake; ADG, average daily weight gain; F/G, feed:gain. ^2^ CON, no fermented cottonseed meal in diet; FCSM, 6% fermented cottonseed meal in diet. ^3^ SEM, standard error of the mean. ^a–b^ Means within a row with different superscripts differ significantly (*p* < 0.05).

**Table 4:** Lipid-related indices in broiler serum, liver, and abdominal fat.

Items ^1^	Treatment ^2^		SEM ^3^	*p*-Value
	CON	FCSM		
Serum				
Glu, mmol/L	12.03 ^a^	10.03 ^b^	0.47	0.04
TG, mmol/L	0.49 ^a^	0.32 ^b^	0.05	<0.01
TC, mmol/L	4.74	4.070	0.22	0.06
NEFA, umol/L	994.10	1006.50	49.67	0.90
HDL-C, mmol/L	1.53	1.25	0.15	0.36
LDL-C, mmol/L	1.35 ^a^	0.92 ^b^	0.11	0.04
Liver				
TG, mmol/gprot	39.01 ^a^	11.72 ^b^	4.34	<0.01
TC, mmol/gprot	0.27	0.26	0.04	0.41
NEFA, umol/gprot	1961.70	1910.70	186.79	0.10
Abdominal fat				
TG, mmol/gprot	139.00	141.70	10.44	0.71
TC, mmol/gprot	0.20	0.19	0.01	0.61
NEFA, umol/gprot	9048.00	8921.22	383.11	0.88

^1^ Glu, glucose; TG, triglyceride; TC, total cholesterol; NEFA, nonesterified free fatty acid; HDL-C, high-density lipoprotein-cholesterol; LDL-C, low-density lipoprotein-cholesterol; gprot, g protein. ^2^ CON, no fermented cottonseed meal in diet; FCSM, 6% fermented cottonseed meal in diet. ^3^ SEM, standard error of the mean. ^a–b^ Means within a row with different superscripts differ significantly (*p* < 0.05).

**Table 5:** Enzyme activity and hormone level in broiler serum, liver, and abdominal fat.

Items ^1^	Treatment ^2^		SEM ^3^	*p*-Value
	CON	FCSM		
Serum				
EPI, ng/mL	11.26 ^b^	16.47 ^a^	0.89	<0.01
GH, ng/mL	9.31 ^b^	12.21 ^a^	0.57	<0.01
INS, mU/L	31.99	35.61	2.49	0.08
Liver				
HSL, U/L	1340.40 ^b^	1981.50 ^a^	110.13	<.001
ME, mIU/L	2231.20 ^a^	1281.90 ^b^	196.23	0.01
ACC, U/mL	937.70	1069.70	98.51	0.07
LPL, U/L	483.60 ^b^	815.20 ^a^	71.12	0.01
FAS, U/mL	1544.90	1551.00	245.01	0.09
EPI, ng/mL	11.64 ^b^	20.18 ^a^	1.59	<0.01
GH, ng/mL	7.88 ^b^	13.66 ^a^	1.18	0.01
Abdominal fat				
HSL, U/L	1310.20 ^b^	1736.70 ^a^	88.17	0.01
ME, mIU/L	1820.83	1832.10	89.99	0.09
ACC, U/mL	895.33	923.33	48.29	0.01
LPL, U/L	592.50 ^b^	773.67 ^a^	34.72	<0.01
FAS, U/mL	1893.00	1916.34	28.52	0.07
EPI, ng/mL	11.26 ^b^	16.47 ^a^	0.891	<0.01
GH, ng/mL	9.31 ^b^	12.21 ^a^	0.568	<0.01

^1^ EPI, epinephrine; GH, growth hormone; INS, insulin; HSL, hormone-sensitive lipase; ME, malic enzyme; ACC, acetyl CoA carboxylase; LPL, lipoprotein lipase; FAS, fatty acid synthase. ^2^ CON, no fermented cottonseed meal in diet; FCSM, 6% fermented cottonseed meal in diet. ^3^ SEM, standard error of the mean. ^a–b^ Means within a row with different superscripts differ significantly (*p* < 0.05).

**Table 6:** Identification of significantly different metabolites in serum between the CON and FCSM groups ^1^.

Superclass	Metabolite Names	Identified by Positive- or Negative-Ion Mode	m/z ^2^	RT ^3^	VIP ^4^	FC (FCSM/CON) ^5^	*p*-Value
Phenylpropanoids	2-Coumarate	negative	163.04	321.99	1.82	0.27	<0.01
Organic acids	2-Ethyl-2-Hydroxybutyric acid	negative	131.07	313.56	1.31	0.74	0.03
Organic acids	2-Hexenal	positive	99.08	4.19	1.94	0.70	<0.01
Organic acids	3-Hydroxybutyric acid	positive	105.05	113.94	1.73	3.38	0.01
Organic acids	Azelaic acid	positive	189.11	393.04	1.81	1.28	<0.01
Organic acids	4-Oxododecanedioic acid	negative	243.12	397.93	1.36	0.41	0.02
Organic acids	Salicylic acid	negative	137.02	405.03	1.47	0.48	0.01
Organic acids	trans-Cinnamic acid	positive	149.06	182.73	2.08	0.70	0.03
amino acid	5-Methylthioadenosine	positive	298.10	230.92	1.53	1.89	0.02
amino acid	N-acetyltryptophan	negative	245.09	362.65	1.43	0.54	0.02
Peptides	Phenylethylamine	positive	122.10	216.64	2.05	0.22	<0.01
Lipids	Docosahexaenoic	positive	329.25	858.32	1.68	1.67	0.01
Pesticides	Choline	positive	104.11	65.25	1.38	0.80	0.04
Terpenoids	Costunolide	negative	231.12	382.28	1.44	0.33	0.02
Steroids	Taurocholic acid	positive	516.30	527.94	1.78	0.25	<0.01
Alkaloids	Indoleacetic	positive	176.07	397.17	1.97	0.31	<0.01
-	Biliverdin	negative	581.24	625.68	1.26	1.64	0.04
-	Dimethylglycine	positive	104.07	133.43	1.73	2.00	0.01
-	Prolylhydroxyproline	positive	229.12	72.91	1.67	1.76	0.01
-	1-heptadecanoyl-sn-glycero-3-phosphocholine	positive	510.35	810.16	1.39	1.69	0.04

^1^ CON, no fermented cottonseed meal in diet; FCSM, 6% fermented cottonseed meal in diet. ^2^ RT, retention time. ^3^ m/z, mass-to-charge ratio. ^4^ VIP, variable importance projection. ^5^ FC, fold change, mean value of peak area obtained from FCSM group/mean value of peak area obtained from CON group. If the FC value is less than 1, it means that metabolites are less in FCSM than in CON.

## Supplementary Materials

The following are available online at https://www.mdpi.com/2076-2615/9/11/930/s1, Table S1: Primers used for quantitative real-time PCR; Table S2: Correlation among fat deposition, lipid-related indices, and predominant serum metabolites; Figure S1: High-resolution LC-MS/MS ion chromatograms of the serum samples corresponding to different diets; Figure S2: Principal component analysis (PCA) of LC-MS/MS of the serum quality control (QC) samples corresponding to different diets; Figure S3: Corresponding validation plots of LC-MS/MS data in positive-ion mode and negative-ion mode between CON and FCSM groups.
