# Supplementary material for: Effect of Fermented Cottonseed Meal on the Lipid-Related Indices and Serum Metabolic Profiles in Broiler Chickens
Source: Animals (Basel). 2019 Nov 7;9(11):930. doi: 10.3390/ani9110930 (PMC6912724; doi:10.3390/ani9110930)
Supplement: Supplementary file 1 [file animals-09-00930-s001.zip › animals-637978 conversion-supplementary/Supplementary_Material.docx]

Supplementary Material

## Supplementary Tables

**Table S1.** Primers used for quantitative real-time PCR.

| **Gene ^1^** | **Primer sequences (5’-3’)** | **Reference sequence** | **Size (bp)** |
| --- | --- | --- | --- |
| *FAS* | F: TCAGGGTGTTCTGGAATGCAA  R: AATCCTGGTGGGCAATCGTAG | NM_205155.2 | 142 |
| *ACC* | F: CTGATGGTCTTTGCC AACTGGA  R: CACGATGTAGGCACCAAACTTGA | NM_205505.1 | 87 |
| *SREBP-1c* | F: TCACCGCTTCTTCGTGGAC  R: CTGAAGGTACTCCAACGCATC | AY029224 | 220 |
| *CPT1* | F: GACAAGTCCTTCACCCTCATCGC  R: GGGTTTGGTTTGCCCAGACAG | NM_013495 | 100 |
| *PPAR-α* | F: TGCACTGGAACTGGATGATAGTGA  R: TCCTACATTTACAAGACCAGGACGA | NM_001001464.1 | 88 |
| *PPAR-γ* | F: TGTGAAGTTCAACGCACTGGAATTA  R: GGAGCTCCAAAGCT TGCAACA | NM_001001460.1 | 146 |
| *HSL* | F: CTCTCCTCATTGGCTTCGGACAAC  R: TGGAGGCGTTGGAGGTGTCTC | NC_006101.5 | 143 |
| *LPL* | F: AGTCAGAGTGAAGT CAGGCGAAAC  R: CTGCTCCAGGCACT TCACAAATA | NM_205282.1 | 115 |
| *β-actin* | F: ATTGTCCACCGCAAATGCTTC  R:AAATAAAGCCATGCCAATCTCGTC | NM_205518.1 | 113 |

^1^ *FAS*, *fatty acid synthase*; *ACC*, *acetyl CoA carboxylase*; *SREBP1c*, *sterol-regulatory element-binding protein 1c*; *CPT1*, *carnitine palmitoyltransferase-1*; *PPAR-α*, *peroxisome proliferator-activated receptor-α*; *PPAR-γ, peroxisome proliferator-activated receptor-γ*; *HSL*, *hormone-sensitive lipase*; *LPL*, *lipoprotein lipase*; *β-actin* an internal reference gene to normalize target gene transcript levels.

## Supplementary Figures

| 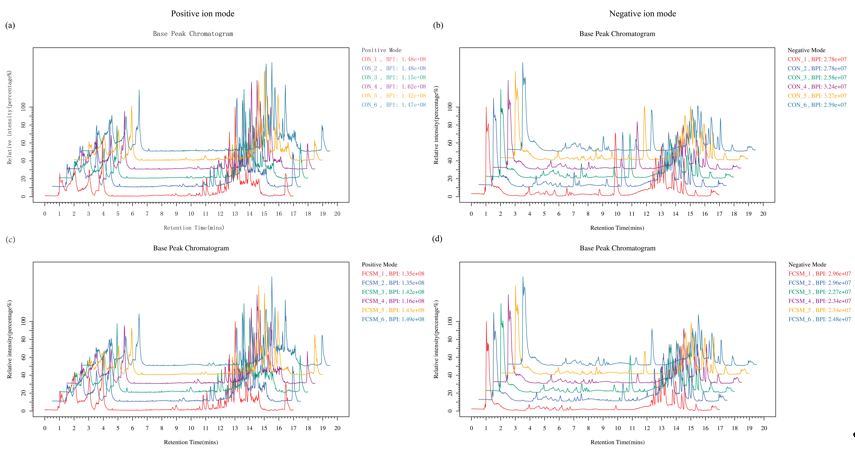 |
| --- |

**Figure S1.** High-resolution LC-MS/MS ion chromatograms of the serum samples corresponding to different diets. CON, no fermented cottonseed meal in diet; FCSM, 6% fermented cottonseed meal in diet. (a) Ion chromatograms of the serum samples in positive ion mode corresponding to CON diets; (b) Ion chromatograms of the serum samples in positive ion mode corresponding to FCSM diets; (c) Ion chromatograms of the serum samples in negative ion mode corresponding to CON diets; (d) Ion chromatograms of the serum samples in negative ion mode corresponding to FCSM diets.

| 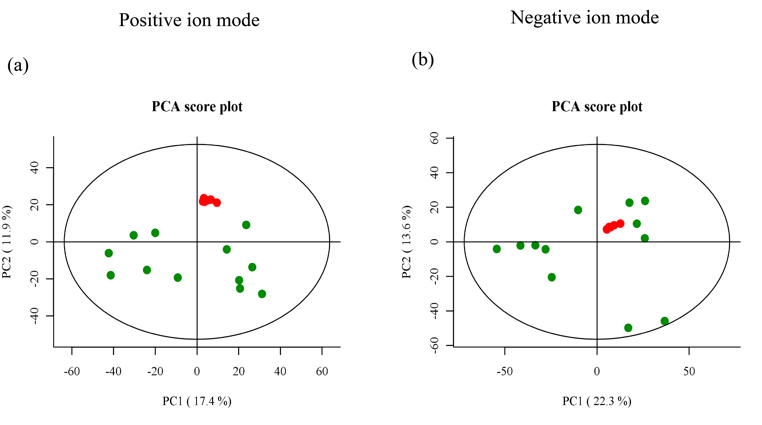 |
| --- |

**Figure S2.** Principal component analysis (PCA) of LC-MS/MS of the serum quality control (QC) samples corresponding to different diets. (a) PCA of QC in positive ion mode. (b) PCA of QC in negative ion mode.

| 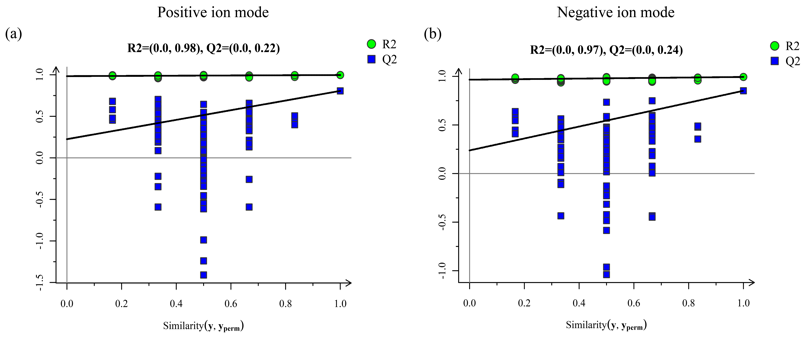 |
| --- |

**Figure 3.** Corresponding validation plots of LC-MS/MS data in positive - ion mode and negative - ion mode between CON and FCSM groups. (a) Corresponding validation plots in positive - ion mode. (b) Corresponding validation plots in negative - ion mode.
